# Shift work, and burnout and distress among 7798 blue-collar workers

**DOI:** 10.1007/s00420-020-01536-3

**Published:** 2020-04-30

**Authors:** Gerben Hulsegge, Willem van Mechelen, Karin I. Proper, Heleen Paagman, Johannes R. Anema

**Affiliations:** 1 Department of Public and Occupational Health, Amsterdam Public Health Research Institute, Amsterdam UMC, Vrije Universiteit Amsterdam, Van der Boechorststraat 7, 1081 BT Amsterdam, The Netherlands; 2 The Netherlands Organization for Applied Scientific Research, TNO, Schipholweg 77–89, 2316 ZL Leiden, The Netherlands; 3 Centre for Nutrition, Prevention and Health Services, National Institute for Public Health and the Environment, Antonie van Leeuwenhoeklaan 9, 3721 MA Bilthoven, The Netherlands; 4 Department Research and Business Development, HumanTotalCare Occupational Health Service, Zwarte Woud 10, 3524 SJ Utrecht, The Netherlands

**Keywords:** Night work, Rotating shift system, Job stress, Work-family conflict, Job satisfaction

## Abstract

**Objective:**

This study aimed to investigate the association between shift work, and burnout and distress, and differences by degree of satisfaction with shift schedule and its impact on private life.

**Methods:**

*Population* 4275 non-shift factory workers and 3523 rotating 5-shift workers. Workers participated between 2009 and 2016 one to three times in the companies’ periodical occupational health checks. Burnout was measured using the distance, exhaustion and competence subscales of the Dutch Maslach Burnout Inventory and distress by the subscale of the Four-Dimensional Symptom Questionnaire (scale: 0–100). Multiple-adjusted linear mixed models were used to assess between- and within-subject associations between shift work and outcomes, and differences by age, years of shift work, and satisfaction with and impact of shift schedule.

**Results:**

Shift work was significantly associated with lower scores on burnout distance (B − 1.0, 95% − 1.8 to 0.3), and among those aged < 48 years with burnout exhaustion (range B − 1.3 to − 1.6). However, the effect sizes were small. Compared to non-shift workers, shift workers dissatisfied with their schedule and those experiencing a high impact on private life had significantly higher burnout (range B 1.7–6.3) and distress levels (range B 4.9–6.1). In contrast, satisfied shift workers and those experiencing a low impact of shift schedule had lower burnout (range B − 0.2 to − 2.2) and no difference in distress levels (*P* ≥ 0.05). No clear pattern by years of shift work was observed.

**Conclusions:**

Shift work was associated with burnout and distress in those who were dissatisfied with or who had perceived high impact on the private life of their shift schedule.

**Electronic supplementary material:**

The online version of this article (10.1007/s00420-020-01536-3) contains supplementary material, which is available to authorized users.

## Introduction

To serve the economic and societal demands of our 24/7 society, it is becoming increasingly necessary for employees to work outside the traditional daytime working hours (US Department of Labor 2008). The increasing number of shift workers causes a growing public health concern, as shift work has been linked with chronic diseases, such as cardiovascular diseases (Vyas et al. 2012). Most previous studies focused on physical health outcomes, while there is a scarcity of studies on the association between shift work and mental health. A few studies indicated that shift work may be associated with mental health outcomes. A disturbed circadian rhythm due to shift work may (1) disrupt neuro-humoral systems (James et al. 2017; Knutsson 2003); (2) increase sleep deprivation (Knutsson 1989, 2003); and (3) produce stressors to private life, such as meeting the demands and expectations from family and friends (Camerino et al. 2010).

Evidence for an association between shift work and mental health is still unclear due to methodological limitations of previous studies and inconsistent findings across studies. Some studies found shift work to be associated with anxiety (Bara and Arber 2009), depression (Bara and Arber 2009), burnout (Jamal 2004; Wisetborisut et al. 2014), and low general mental health (Barnes-Farrell et al. 2008), while others could not detect such associations (Berthelsen et al. 2015; Jamal and Baba 1997; Kawabe et al. 2015; Soric et al. 2013), or even found favorable effects of shift work (Nabe-Nielsen et al. 2011). The abovementioned studies may be biased since they did not take basic confounders into account, such as age and gender. In addition, to our knowledge, earlier studies on this topic lacked detailed insight into shift work (e.g. type of shift schedule) and duration of shift work, while the type of shift work and the cumulative exposure to shift work presumably affect health (Stevens et al. 2011). Thus, it is needed to investigate differences in mental health between non-shift workers and shift workers with homogeneous shift schedules (e.g. fast-forward rotating shift schedule), and to clarify differences by years of exposure to shift work.

According to the job-demand resources theory, Burnout is considered to be caused by long-term exposure to high job demands (e.g. time pressure) in which these demands exceed the individual’s adaptive resources (e.g. job control, social support) (Demerouti et al. 2001). Stress may also occur when individual resources deplete due to situations outside work, such as difficulty to balance work and family activities (Grandy and Cropanzano 1999; Hobfoll 1989). In addition, it has been shown that high job satisfaction protects against the long-term effects of stress on burnout (Kalliath and Morris 2002; Visser et al. 2003). We hypothesize that these findings of work-family balance and job satisfaction are also applicable to the shift schedule. We hypothesize that satisfaction with shift schedule and perceived impact of the schedule on private life activities moderates the association between shift work and distress and burnout. Shift workers with low satisfaction about their shift schedule may experience irregular shifts as a recurrent stressor, causing distress and eventually exhaustion, whereas this might not be the case in those with high schedule satisfaction. In addition, two studies observed shift work to be associated with work-family conflict (Camerino et al. 2010; Tuttle and Garr 2012), which in turn was associated with burnout in one of those studies (Camerino et al. 2010). Thus, shift work satisfaction and shift work-related work-private life balance may play a role in the association between shift work and burnout, but this has yet to be confirmed.

Therefore, the aim of this study was to investigate the associations between shift work, including the number of years worked in shifts, and burnout and distress as measures of mental health, in a large homogeneous population of factory workers. Our second aim was to investigate whether these associations differ by degree of satisfaction with shift schedule and the perceived impact of shift schedule on private life.

## Methods

### Population

A nationwide Dutch occupational health care service has continuously gathered data about work and health using standardized questionnaires and physical examinations, as part of their standard periodical occupational health checks. Workers from two large industrial companies were invited to voluntarily take part in this health check, approximately once every 3 years, between 2009 and 2017. In total, 9614 workers participated in at least one health check. In 2018, the questionnaire data were enriched by company records on shift work history. Participants without company records on shift work (*n* = 1307), ex-shift workers at study inclusion (*N* = 329), 2-shift workers due to the relatively few workers with this specific shift schedule (*N* = 141), or those with missing data on burnout and/or distress (*N* = 16) or covariates (*N* = 23) were excluded. The included workers worked in manufacturing companies, for example, as manufacturing and assembly workers, operators, team/shift leaders, and office worker. The Medical Ethics Committee of the VU University Medical Center Amsterdam approved the study.

### Shift work

The Human Resources department of the two companies provided for each worker information on the type of shift work, i.e. 2-shift work, 5-shift work, and non-shift work. Non-shift workers were categorized as office workers and day workers. No information was available on whether the shift workers were office workers or not. The 5-shift schedule consisted of a fast-forward rotating schedule of two-morning shifts from 6:00 to 14:00, two-afternoon shifts from 14:00 to 22:00, and two-night shifts from 22:00 to 6:00, followed by three or four days off work. For each worker, the start date of engaging employment in shift work at these two companies was provided by the companies and was used to calculate the number of years of exposure to shift work.

### Burnout and distress

Burnout was measured using the Dutch version of the Maslach Burnout Inventory, i.e. the validated 16-item Utrecht BurnOut Scale (UBOS) (Schaufeli and Van Dierendonck 2000). The UBOS measures three burnout dimensions: emotional exhaustion (eight items), mental distance or depersonalization (five items), and competence or personal accomplishment (seven items). An example of an item of the emotional exhaustion scale is “I feel emotionally drained by my work”. An example of a mental distance item is “I worry that this job is hardening me emotionally”. An example of the competence scale is “I have accomplished many useful things in my work”. All items were scored on 7-point scales from 0 (‘never’) to 6 (‘always’). The items of each scale were summed and transformed to a scale ranging from 0 to 100 with higher scores indicating burnout. Previous studies indicated that the internal consistencies, factorial and construct validity of the UBOS are satisfactory (Leiter and Schaufeli 1996; Schutte et al. 2000; Taris et al. 1999). Distress was measured with the validated 16-item distress subscale of the Four-Dimensional Symptom Questionnaire (Terluin et al. 2006). Each item was rated on a 5-point scale ranging from ‘no’ to ‘very often or constantly’ and refers to symptoms experienced during the past week. All items were summed and transformed into a score ranging from 0 to 100 with higher scores indicating distress.

### Shift work features

Shift work features have been measured in the last three years of the health checks and were, therefore, only available for a subsample of the shift workers (*N* = 1428, 41%). Satisfaction with work schedule was measured with the following statement: “I am satisfied with working in shifts, rotating shifts or irregular work times”. Response options were given on a 5-point Likert scale. For the analysis, this variable was dichotomized into satisfied (‘satisfied’, ‘very satisfied’ and ‘neutral’) and dissatisfied (‘not satisfied at all’, ‘not satisfied’). Impact of shift work on private life was measured by calculating the average of the scores on the following four statements (Cronbach’s *α* 0.85): “My work schedule offers me enough opportunity to perform (1) tasks/obligations at home; (2) hobbies and interests; (3) social activities, such as meeting with family, friends and acquaintances; and (4) sports. Answer options ranged from very much (score ‘0’) to very little (score ‘4’) on a 5-point scale. This variable was dichotomized into low impact on private life (score ≤ 2) and high impact on private life (score > 2).

### Covariates

Age (continuous), gender (men vs. women), education (low vs. intermediate vs. high), marital status (married/living together vs. single), children living at home (yes vs. no/not applicable), and working hours/week were measured by single questions. Workplace social support was measured by six items (Cronbach’s *α* 0.87), with a 5-point Likert scale about support from supervisor and colleagues. These items are part of the validated questionnaire on psychosocial work demands (Veldhoven van and Meijman 1994). Work pressure was measured with 5 items (Cronbach’s *α* 0.87) of the questionnaire of Veldhoven and Meijman (1994) on 5-point Likert scales (Veldhoven van and Meijman 1994). The items on workplace social support and work pressure were summarized into two continuous scores for the analyses.

### Data analysis

Linear mixed models were used to examine the association between shift work (non-shift worker vs. 5-shift workers), and. burnout and distress. As all outcome measures at the different time-points were analyzed together, the overall results represent the combination of within-subject (effect of the longitudinal change from shift worker to non-shift worker, and vice versa) and between-subject (cross-sectional comparison between shift workers and non-shift workers) associations. We also used mixed models to examine the dose-exposure association between the number of years of shift work, and burnout and distress, by comparing shift workers who had worked for < 5 years, 5–9 year, 10–14 years, 15–19 years, 20–24 years, and ≥ 25 years in irregular shifts with non-shift workers. We also stratified shift workers with low and high satisfaction according to shift schedule and compared their burnout and distress scores with non-shift workers. Similarly, we stratified shift workers into those who had experienced a low or high impact of their shift work schedule on private life and compared their burnout and distress scores with non-shift workers. All analyses were adjusted for age, gender (model 1), educational level, marital status, children living at home, working hours/week, workplace social support, and work pressure (model 2). We also examined differences by age, using interaction terms between shift work and age. When significant (*P* < 0.05), analyses were stratified into quartile groups based on age. In a sensitivity analysis, we excluded non-shift workers with office work, to make the non-shift workers more comparable to shift workers as we assumed that only few shift workers were office workers.

## Results

### Descriptive statistics

Of the 7798 participants, 4248 participated in one, 3422 in two and 128 in three waves. Of the shift workers who had participated in two or three waves, 98.7% remained shift worker during follow-up, and 1.3% became non-shift worker. Only 0.7% of the non-shift workers became a shift worker during follow-up.

At inclusion into the study, shift workers were on average 45.8 years (SD 10.9) and non-shift workers 44.6 years (SD 11.3) (Table 1). Most participants were male, i.e. 97% and 85% of the shift and non-shift workers, respectively. Shift workers (29%) were more often low educated compared to non-shift workers (7%). Shift workers worked at study inclusion on average 17.5 years (SD 8.9) in shifts. Characteristics of the subsample of shift workers with information on shift work features (*N* = 1428) did not substantially differ from the total group of shift workers (*N* = 3523).

**Table 1:** Characteristics at study inclusion

	Non-shift workers *N* = 4275	5-shift workers *N* = 3523	Subsample 5-shift workers with information on shift work features *N* = 1428
Demographics			
Age (years)	45.8 ± 10.9	44.6 ± 11.3	43.8 ± 10.8
Gender (male)	3634 (85%)	3424 (97%)	1375 (96%)
Educational level			
Low	306 (7%)	1,033 (29%)	343 (24%)
Intermediate	1489 (35%)	2313 (66%)	1008 (71%)
High	2480 (58%)	170 (5%)	77 (5%)
Marital status			
Married/living together	3246 (76%)	2537 (72%)	1021 (72%)
Living alone	855 (20%)	780 (22%)	315 (22%)
Other	174 (4%)	206 (6%)	92 (6%)
Living with children	2373 (56%)	1837 (52%)	798 (56%)
Years worked in shifts	NA	17.5 ± 8.9	16.4 ± 9.3
Working hours per week	38.8 ± 6.2	34.4 ± 3.9	34.5 ± 3.9
Work pressure (scale 0–4)	2.8 ± 0.8	2.5 ± 0.8	2.6 ± 0.8
Workplace social support (scale 0–4)	3.8 ± 0.8	3.7 ± 0.8	3.7 ± 0.8
Burnout exhaustion (scale 0–100)	20.7 ± 14.6	21.2 ± 15.4	20.7 ± 14.8
Burnout distance (scale 0–100)	19.7 ± 15.9	19.5 ± 16.8	18.4 ± 16.5
Burnout competence (scale 0–100)	30.1 ± 14.0	32.3 ± 16.6	31.6 ± 16.4
Distress (scale 0–100)	22.7 ± 22.1	25.1 ± 24.2	23.3 ± 24.9

Data are presented as means ± standard deviations and as numbers and (percentages) and (numbers)

Burnout was measured with the Dutch Maslach Burnout Inventory; higher scores indicating burnout. Distress was measured with the Four-Dimensional Symptom Questionnaire; higher scores indicating distress

*NA* not applicable

### Shift work, and burnout and distress

Burnout scores ranged from 19.5 to 32.3 in shift workers and from 19.7 to 30.1 in non-shift workers (Table 1). The mean distress score was 25.1 in shift workers and 22.7 in non-shift workers. In age- and gender-adjusted models, 5-shift work was significantly associated with higher burnout exhaustion (B 0.7, 95% CI 0.01 to 1.3), burnout competence (B 1.9, 95% CI 1.2 to 2.5), and distress (B 3.0, 95% CI 2.0 to 4.0) (Table 2). In fully adjusted models, all associations became non-significant (*P* < 0.05), and 5-shift work was significantly associated with lower burnout exhaustion (− 0.9, 95% CI − 1.6 to − 0.2) and burnout distance (B − 1.0, 95% CI − 1.8 to − 0.3).

**Table 2:** Linear regression coefficients for differences in burnout and distress between 5-shift workers (*N* = 3523) and non-shift workers (*N* = 4275) (reference)

	Regression coefficient (95% confidence interval)			
	Burnout exhaustion	Burnout distance	Burnout competence	Distress
Model 1	0.7 (0.01 to 1.3)*	**−** 0.1 (**−** 0.8 to 0.6)	**1.9 (1.2 to 2.5)**	3.0 (2.0 to 4.0)*
Model 2	**−** **0.9 (−** **1.6 to − 0.2)**	**−** **1.0 (−** **1.8 to − 0.3)**	**−** 0.4 (**−** 1.2 to 0.3)	1.0 (**−** 0.1 to 2.2)

Model 1: adjusted for age, gender

Model 2: education, marital status, living with children, working hours/week, work pressure, and workplace social support

Boldface indicates statistical significance (*P* < 0.05)

Burnout was measured with the Dutch Maslach Burnout Inventory; higher scores indicating burnout. Distress was measured with the Four-Dimensional Symptom Questionnaire; higher scores indicating distress

^*^Significant interaction with age (interaction *P* value < 0.05)

The association between shift work and burnout exhaustion and distress differed significantly by age (interaction *P* < 0.05), with shift work with increasing age being more negatively associated with burnout. Shift work was significantly associated with lower burnout exhaustion among those aged ≤ 48 years (range B − 1.3 to − 1.6), while this was not the case among those aged > 48 years (Table 3). Shift work was not significantly associated with distress in any of the age groups. Although we observed no clear dose-exposure association between the number of years of shift work, and burnout and distress, the positive association between 5-shift work and burnout distance was only present among those who had worked in shifts for 0–4 years (B − 4.5, 95% CI − 6.0 to − 3.1) and 5–9 years (B − 2.0, 95% CI − 3.3 to − 0.7) (Supplemental Table 1).

**Table 3:** Linear regression coefficients for differences in burnout exhaustion and distress between 5-shift workers (*N* = 3523) and non-shift workers (*N* = 4275) (reference) stratified by age group

	Outcome	
	Regression coefficient (95% confidence interval)	
	Burnout exhaustion	Distress
< 40 years	**−** **1.3 (−** **2.7 to − 0.02)**	0.1 (**−** 2.1 to 2.2)
40–48 years	**−** **1.6 (−** **3.0 to − 0.2)**	1.2 (**−** 1.1 to 3.6)
49–55 years	**−** 1.2 (**−** 2.6 to 0.1)	0.3 (**−** 1.7 to 2.4)
> 55 years	0.3 (**−** 1.4 to 1.9)	1.4 (**−** 1.0 to 3.8)

Linear mixed models adjusted for age, gender, education, marital status, living with children, working hours/week, work pressure, support supervisor and colleagues

Boldface indicates statistical significance (*P* < 0.05)

Burnout was measured with the Dutch Maslach Burnout Inventory; higher scores indicating burnout. Distress was measured with the Four-Dimensional Symptom Questionnaire; higher scores indicating distress

Note: other burnout outcomes not presented as they did not significantly differ by age (interaction *P* value ≥ 0.05)

### Shift work features, and burnout and distress

Shift workers who were dissatisfied with their shift schedule had significantly higher scores on all burnout scales (range B 3.6 to 6.3) and distress (B 6.1, 95% CI 3.4 to 8.8), compared to non-shift workers (Table 4). In contrast, shift workers who were satisfied with their shift schedule had significantly lower scores on burnout distance and competence (range B − 1.8 to − 0.8) and did not significantly differ in burnout exhaustion and distress (*P* ≥ 0.05). Similarly, shift workers who had perceived their shift schedule to have a high impact on commitments/sports/hobbies had significantly higher scores on all burnout dimensions (range B 1.7 to 3.8) and distress (B 4.9, 95% CI 3.2 to 6.6) than non-shift workers. The shift workers who had perceived the low impact of their shift schedule on their private life had significantly lower scores on all three burnout scales (range B − 2.2 to − 0.9). Most associations did not differ by age (interaction *P* ≥ 0.05) (Table 4 and Supplemental Table 2).

**Table 4:** Linear regression coefficients for differences in burnout and distress between non-shift workers (4275) (reference) and a subsample of 5-shift workers (1428) stratified by satisfaction with shift schedule and impact of shift schedule on private life

		Regression coefficient (95% confidence interval)			
		Burnout exhaustion	Burnout distance	Burnout competence	Distress
Satisfaction with shift schedule					
Non-shift worker		ref	ref	ref	ref
Satisfied shift workers	Model 1	0.1 (**−** 0.7 to 0.8)	**−** **1.5 (−** **2.4 to − 0.7)**	0.1 (**−** 0.7 to 0.8)	1.0 (**−** 1.9 to 2.1)
	Model 2	**−** 0.2 (**−** 0.9 to 0.5)	**−** **1.8 (−** **2.7 to − 1.0)**	**−** **0.8 (−** **1.5 to − 0.1)**	0.3 (**−** 0.8 to 1.5)
Dissatisfied shift workers	Model 1	**7.7 (5.8 to 9.5)**	6.7 (4.8 to 9.0)*	**5.1 (3.3 to 6.9)**	**8.3 (5.5 to 11.1)**
	Model 2	**6.3 (4.6 to 8.0)**	**5.2 (3.2 to 7.1)**	**3.6 (1.9 to 5.3)**	**6.1 (3.4 to 8.8)**
Impact shift schedule on commitments/sport/hobby’s					
Non-shift worker		ref	ref	ref	ref
Shift workers experiencing no impact	Model 1	**−** **1.1 (−** **1.9 to − 0.2)**	**−** **2.5 (−** **3.5 to − 1.5)**	**−** **1.2 (−** **2.1 to − 0.4)**	**−** 0.8 (**−** 2.1 to 0.5)
	Model 2	**−** **0.9 (−** **1.7 to − 0.1)**	**−** **2.2 (−** **3.1 to − 1.2)**	**−** **1.5 (−** **2.3 to − 0.6)**	**−** 0.8 (**−** 2.1 to 0.5)
Shift workers experiencing impact	Model 1	**5.3 (4.1 to 6.4)**	**3.8 (2.4 to 5.1)**	4.5 (3.3 to 5.7)*	**7.3 (5.5 to 9.1)**
	Model 2	**3.8 (2.7 to 4.9)**	**1.7 (0.4 to 2.9)**	**2.7 (1.6 to 3.8)**	**4.9 (3.2 to 6.6)**

Model 1: adjusted for age, gender

Model 2: education, marital status, living with children, working hours/week, work pressure, and workplace social support

Boldface indicates statistical significance (*P* < 0.05)

Burnout was measured with the Dutch Maslach Burnout Inventory; higher scores indicating burnout. Distress was measured with the Four-Dimensional Symptom Questionnaire; higher scores indicating distress

^*^Significant interaction with age (interaction *P* value < 0.05)

### Sensitivity analysis

Exclusion of office workers did not change the results (data not shown).

## Discussion

In the total population, we observed no differences in burnout and distress between shift and non-shift workers, except for lower burnout distance and, only among those aged < 48 years, burnout exhaustion among shift workers. We additionally showed that there was no clear association between the number of years in shift work, and burnout and distress. However, shift workers dissatisfied with their shift schedule or who had perceived a high impact of their schedule on private life had higher burnout and distress levels than non-shift workers.

The difference in burnout distance and exhaustion between shift and non-shift workers was statistically significant, but not meaningful as they differed only by 1 point on a 0–100 scale. In line with our findings, studies found shift and non-shift workers to have no difference in perceived stress in police officers (Gerber et al. 2010), psychological health (i.e. negative feelings, self-esteem) in nurses (Soric et al. 2013), and depression, anxiety, and burnout in health care workers (Berthelsen et al. 2015; Jamal and Baba 1997). We expected shift work to increase the risk of distress and burnout due to disruption of the circadian rhythm, sleep problems, and stress to private life (Camerino et al. 2010; Knutsson 1989, 2003). The reasons why the present study observed no meaningful association between shift work and burnout and distress in the total population are unclear. It may be that selection mechanisms have played a role since workers with sleep problems and those experiencing a mismatch between work-time preferences and non-day work less often start shift work or tend to leave shift work, i.e. healthy worker effect (Knutsson and Åkerstedt 1992; Nabe-Nielsen et al. 2008, 2010). It may also be that those with burnout and distress problems had left shift work, which may have led to some underestimation of the association between shift work, and burnout and distress. In addition, shift work may be preferred by some workers as it fits well in their life’s or gives other benefits, such as higher wages. According to the effort-reward-imbalance model and the job-demand resources theory (Demerouti et al. 2001; Siegrist 1996), for these workers the rewards of shift work may outweigh the load of shift work, Finally, the negative effects of shift work were perhaps buffered by the availability of resources in shift workers, such as job control (Demerouti et al. 2001).

This buffering effect of resources and rewards is supported by our findings of differences by satisfaction with and impact on the private life of the shift schedule in the association between shift work, and burnout and distress. Shift workers dissatisfied with their shift schedule or experiencing a high impact of their schedule on private life had more burnout and distress complaints than non-shift workers, whereas the opposite was true for shift workers satisfied or experiencing a low impact on private life. Several mechanisms may explain why satisfaction and impact of schedule moderates the effects of shift work, burnout and distress. It may be that shift workers who were dissatisfied with their schedule may have had fewer resources to buffer the effect of shift work. One study observed a lack of supervisors support to contribute to dissatisfaction with shift schedule (Beutell 2010), indicating a lack of resources to adapt to shift work. In contrast, those satisfied with their schedule may have deliberately chosen to work in shifts and thereby may have experienced more job control or more satisfaction about work times and higher wages (Fenwick and Tausig 2001), which also may have buffered the negative effects of shift work. Dissatisfied shift workers may also have had more problems with coping with their schedule than satisfied workers and have been found to experience more sleep problems (Axelsson et al. 2004), which may contribute to distress and burnout symptoms (Söderström et al. 2012). A review also showed that lack of job satisfaction was associated with mental health problems, including (work-related) stress and burnout (Faragher et al. 2005). In addition, work-family imbalance may explain why shift workers being dissatisfied and those experiencing a high impact of schedule on private life had more burnout and distress complaints. A study in Italian nurses and a study on a nationally representative sample of workers showed shift work to be associated with more work-family conflict, which was defined by demands of work interfering with family life and home, or having enough time for family and other important people in life (Camerino et al. 2010; Tuttle and Garr 2012). The consequently reduced opportunity to recover from job demands and an irregular work schedule may contribute to distress and eventually burnout (Lingard and Francis 2005). This line of reasoning is also supported by a review of Allen et al. (2000), which showed evidence for a relationship between work-family imbalance and mental health problems, including burnout. Finally, it should be noted that due to the cross-sectional nature of our study, distress or burnout symptoms may also have led to less satisfaction with or more impact on the private life of shift schedule among shift workers.

Thus, several factors may contribute to distress and burnout symptoms among shift workers dissatisfied with and experiencing a high impact on the private life of their shift schedule. The observed differences of 3–6 points correspondent to a hedges’ g (i.e. a measure of effect size) of up to 0.43, which implies that the differences between groups were small to medium. Although differences in burnout and distress scores seem small on an individual level, such differences are, as eloquently explained by Geoffrey Rose (Grandy and Cropanzano 1999), still likely to contribute to the burden of burnout and distress on a population level. The observed association between shift work and burnout and distress problems among dissatisfied shift workers and those experience high impact of their schedule on private life may especially be relevant considering the high prevalence of burnout and distress complaints among workers, leading to high morbidity and costs (Organisation for Economic Cooperation and Development 2012). Thus, the results of our study highlight the importance to further explore the relation between shift work, and burnout and distress, and the role of shift schedule satisfaction and impact of the schedule on private life.

The strength of the present study is the use of a large homogeneous group, which minimizes residual confounding related to differences across companies, such as work tasks and the organizational culture of companies. Shift work schedule and history of shift work were determined using objective registry data. It is often argued to use change scores to analyze prospective relations. However, the error of change scores are large (Cain et al. 1992; Twisk and Proper 2004), and may even be larger than the between-subject difference. We, therefore, used a model in which we compared levels of distress and burnout symptoms at several time points, and its association with shift work status (within an individual). Unfortunately, only few workers changed from being shift worker to non-shift worker, or vice versa, during follow-up. The presented results are, therefore, mainly the result of a cross-sectional between-person comparison, and one should be cautious when interpreting the temporal association between shift work, and burnout and distress. Satisfaction and impact of shift schedule have been measured in a subsample (41%) of the shift workers. As characteristics did not differ between this subgroup and the total group of shift workers at study inclusion, we expect this to have had little impact on the results. The questions on shift work satisfaction and impact on private life were specifically developed for shift workers and consequently not measured in non-shift workers. We are, therefore, unaware of job satisfaction and work-private balance of non-shift workers and their relationship with burnout and distress in this group. Formal moderation analyses were, therefore, not possible. To have a more comparable reference category than the non-shift workers, we also analyzed within shift workers the associations between shift work satisfaction and impact on private life with burnout and distress. This resulted in the comparable association as presented (data not shown), i.e. low satisfaction with and high impact of shift schedule on private life were associated with more burnout and distress complaints among shift workers. Another limitation of the questions on shift work satisfaction and impact on private life is that they have not been validated. Future studies are, therefore, needed to validate these questions, or a study needs to be conducted using validated questionnaires of general measures (not related to shift work) of job satisfaction and work-private balance. Dichotomization of the work-private balance score was based on the distribution of the data. Although the conclusions are unlikely to change, changing the cut-off value to a worse or better life-balance score is likely to result in a slightly larger or smaller difference in distress and burnout between shift and non-shift workers, respectively. Although the production industry represents a large part of the shift work population, generalization of the results is limited to industrial workers with rotating shift schedules. As described above, workers who experience burnout and distress complaints are probably more likely to leave shift work than workers without burnout and distress complaints, which can cause a healthy worker effect. This common methodological problem of shift work research may have resulted in an underestimation of the association between shift work and burnout and distress (Nabe-Nielsen et al. 2008). Finally, due to the cross-sectional nature of the present study and the lack of knowledge on the relevance of the observed differences it is not possible to make strong practical recommendations. Longitudinal studies are necessary to confirm our findings. If our findings can be replicated dissatisfied shift workers and those experiencing a high impact of their schedule on private life may need special attention in the prevention of burnout and distress. It may then be useful to implement interventions to reduce work-family conflict (Camerino et al. 2010; Wilson et al. 2007), increase schedule control (Tuttle and Garr 2012), and educate managers, shift workers and their spouses to improve social support and coping strategies to buffer the negative effects of shift work (Knauth and Hornberger 2003).

In conclusion, shift work was not associated with an increased risk of burnout and distress in the total population. However, shift workers who were dissatisfied with their schedule or who had experienced a high impact of their schedule on private life had more distress and burnout complaints compared to non-shift workers, whereas satisfied shift workers or those who had experienced a low impact on private life had less burnout and distress complaints. These findings imply that prevention of burnout and distress may need to have special attention for shift workers who are dissatisfied with their schedule or perceive a large work-life imbalance due to their irregular schedule.

## Electronic supplementary material

Below is the link to the electronic supplementary material.

Supplementary file1 (DOCX 26 kb)

## Data Availability

Due to ethical restrictions related to participant consent and high sensitivity of the company data, all relevant data are under the conditions of HumanTotalCare available upon request to the responsible senior manager Research & Business Development at HumanTotalCare: Heleen Paagman (email: heleen.paagman@arboned.nl).
