# Supplementary material for: Shift work, and burnout and distress among 7798 blue-collar workers
Source: Int Arch Occup Environ Health. 2020 Apr 30;93(8):955–63. doi: 10.1007/s00420-020-01536-3 (PMC7519910; doi:10.1007/s00420-020-01536-3)
Supplement: Supplementary file 1 — Supplementary file1 (DOCX 26 kb) [file 420_2020_1536_MOESM1_ESM.docx]

**Supplemental Table 1**. Linear regression coefficients for differences in burnout and distressbetween non-shift workers (N=4275) (reference) and 5-shift workers (N=3523) stratified by number of years exposure to shift work.

|  | Regression coefficient (95% confidence interval) | | | |
| --- | --- | --- | --- | --- |
|  | **Burnout exhaustion** | **Burnout distance** | **Burnout competence** | **Distress** |
| Non-shift workers | ref | ref | ref | Ref |
| Shift work 0-4 years | **-2.7 (-4.0 to -1.4)** | **-4.5 (-6.0 to -3.1)** | **-1.9 (-3.3 to -0.5)** | -1.5 (-3.6 to 0.7) |
| Shift work 5-9 years | -0.7 (-1.9 to 0.5) | **-2.0 (-3.3 to -0.7)** | -0.3 (-1.5 to 0.9) | **2.1 (0.2 to 3.9)** |
| Shift work 10-14 years | -0.1 (-1.5 to 1.3) | -0.1 (-1.7 to1.4) | -0.9 (-2.4 to 0.5) | 1.4 (-0.8 to 3.6) |
| Shift work 15-19 years | -0.1 (-2.1 to 1.9) | 1.2 (-1.1 to 3.4) | -0.04 (-2.1 to 2.0) | -1.7 (-4.8 to 1.5) |
| Shift work 20-24 years | **-1.1 (-2.0 to -0.1)** | -0.4 (-1.4 to 0.6) | -0.1 (-1.0 to 0.8) | 0.5 (-0.9 to 1.9) |
| Shift work ≥25 years | -0.2 (-1.2 to 0.7) | -0.7 (-1.7 to 0.3) | -0.04 (-1.0 to 0.9) | 1.4 (-0.1 to 2.8) |

Linear mixed models adjusted for age, gender, education, marital status, living with children, working hours/week, work pressure, support supervisor and colleagues.

Boldface indicates statistical significance (*P* < 0.05).

Burnout was measured with the Dutch Maslach Burnout Inventory; higher scores indicating burnout. Distress was measured with the Four Dimensional Symptom Questionnaire; higher scores indicating distress.

**Supplemental Table 2**. Linear regression coefficients for differences in burnout and distress between non-shift workers (4275) (reference) and a subsample of 5-shift workers (1485) stratified by age group and either by satisfaction with or impact of shift schedule.

|  | Regression coefficient  (95% confidence interval) | | |
| --- | --- | --- | --- |
|  | **Burnout distant** | | **Burnout competence** |
|  | **Dissatisfied shift workers vs non-shift workers** | | **Shift workers experiencing high impact of schedule on private life vs non-shift workers** |
|  | |  |  |
| <40 years | 0.5 (-3.8 to 4.8) | | 0.6 (-1.6 to 2.8) |
| 40-48 years | **4.9 (0.7 to 9.1)** | | 0.1 (-2.2 to 2.5) |
| 49-55 years | **4.6 (1.0 to 8.1)** | | **4.6 (2.5 to 6.8)** |
| >55 years | **10.2 (6.0 to 14.3)** | | **5.2 (2.6 to 7.8)** |

Linear mixed models adjusted for age, gender, education, marital status, living with children, working hours/week, work pressure, support supervisor and colleagues.

Boldface indicates statistical significance (*P* < 0.05).

Burnout was measured with the Dutch Maslach Burnout Inventory; higher scores indicating burnout. Distress was measured with the Four Dimensional Symptom Questionnaire; higher scores indicating distress.
